# Topology‐Orchestrated Multi‐physical Energy Control in Turtle Shell‐Inspired Metamaterials

**DOI:** 10.1002/adma.74125

**Published:** 2026-07-16

**Authors:** Xi Wang, Shiyi Wang, Bingzhi Chen, Xinwei Li

**Affiliations:** ^1^ Key Laboratory of Railway Industry on Safety Service Key Technologies for High‐speed Train Zhan Tianyou Honors College Dalian Jiaotong University Dalian China; ^2^ Newcastle University in Singapore Faculty of Science Agriculture, and Engineering Newcastle University Newcastle upon Tyne UK

**Keywords:** bioinspiration, energy absorption, lattice structure, metamaterial, sound absorption

## Abstract

Achieving metamaterials that are simultaneously mechanically, acoustically, and thermally functional remains a central challenge in architected material design. In this research, we report a turtle shell bioinspired multifunctional lattice metamaterial (BMLM) that overcomes this limitation through topology‐driven hybrid‐coupling of distinct dissipation and transport pathways within a single hierarchical framework. The integration of arcuate plate‐strut geometries with engineered microporosity enables coordinated mechanical energy absorption, acoustic attenuation, and convective thermal transport. Experimentally validated, the proposed architecture achieves an exceptional specific compressive energy absorption of 55.7 kJ/kg alongside broadband acoustic performance, exhibiting an average sound absorption coefficient of 0.954 and near‐unity absorption (>0.9) sustained continuously from 2150 to 4250 Hz. Notably, the structure maintains acoustic stability under severe deformation; even at 40% compressive strain, absorption remains above 0.9 across a 2.1 kHz bandwidth, demonstrating deformation‐resilient functionality. Moreover, the interconnected open‐cell topology facilitates efficient airflow and convective thermal dissipation through its high surface‐area‐to‐volume ratio, comparing superior to standard plate‐fin heat sinks. Collectively, this work establishes a topology‐driven strategy for designing bio‐inspired metamaterials with robust, cross‐domain performance.

## Graphical abstract

A turtle shell‐inspired lattice metamaterial efficiently manages acoustic, fluid‐thermal, and mechanical energy flows simultaneously. The unique hybrid‐coupling architecture achieves superior sound attenuation via pore‐cavity resonance, facilitates effective airflow transport, and maximizes mechanical energy absorption through multistage cell deformation. This bioinspired multifunctional design provides a comprehensive strategy for engineering applications requiring multidomain physical performance.

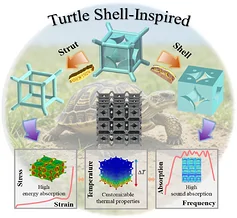

## 1 Introduction

Biomimetic design strategies have emerged as a paradigm shift for architected materials, offering robust solutions to complex societal and engineering challenges. The proliferation of additive manufacturing has revolutionized this field, unlocking an unprecedented design space for realizing intricate, bioinspired 3D topologies at the macroscale [1, 2, 3]. Nature provides a rich library of sophisticated heterogeneous architectures, exemplified by the hierarchical septum‐wall architecture of cuttlebone [4], hierarchically microstructure in coral fungus [5], dual‐gradient cellular structure in sunflower stem [6], and double‐diagonal reinforcement configuration found in deep‐sea glass sponges [7]. However, while existing bioinspired designs have achieved superior mechanical properties, they predominantly suffer from a mono‐functional focus. Bridging this gap is critical, as modern engineering systems increasingly demand architectures capable of the synergistic manipulation of acoustic, thermal, and mechanical loads.

In early engineering structural designs, load‐bearing and sound‐absorbing functions were long segregated. Sound absorption mainly relied on porous materials such as glass fiber and polyurethane foam [8], while load‐bearing functions were realized by metal plates, composite laminates, and similar materials. The core contradiction at this stage lay in the fact that sound‐absorbing materials possessed weak mechanical properties, with low stiffness and strength, rendering them unsuitable as primary load‐bearing components. Conversely, high‐strength load‐bearing structures, such as metal honeycomb panels, had limited sound absorption modulation capabilities due to a lack of pore design. This functional separation led to structural redundancy, making it difficult to meet the multiple demands for load‐bearing and noise reduction in fields such as aerospace and rail transportation. Subsequently, with the rise of composite materials and multidisciplinary optimization technologies, researchers attempted to preliminarily integrate sound‐absorbing and load‐bearing functions through material compounding and structural design.

Representative directions include the following three categories: First, metal foam composites. These utilize the pore structure of open‐cell metal foams to achieve sound absorption, while employing a metal skeleton to enhance mechanical performance; however, they suffer from a narrow sound absorption frequency band (mainly targeting medium‐to‐high frequency noise) and limited load‐bearing strength. Second, sandwich structure design. This involves combinations such as corrugated plates [9], or honeycomb cores with perforated face sheets [10]. Through core configuration design, these structures balance in‐plane load‐bearing and sound wave dissipation, yet their sound absorption efficiency remains low. Third, gradient impedance structures. These optimize acoustic impedance matching through the gradient distribution of material density or porosity [11], but the theory for the synergistic design of mechanical and acoustic properties is not yet mature. While these methods have improved the comprehensive performance of structures to a certain extent, they still face issues such as narrow absorption bands and unclear multifunctional synergistic mechanisms. In particular, current deep synergistic design theories offer limited guidance regarding mechanical and acoustic properties. Thereafter, some scholars utilized the concept of decoupling to assemble structural elements with single functions into composite structures; this method is termed the weak coupling design method. Based on the Helmholtz resonator principle, similar structural design forms include cuttlebone‐inspired micro‐structured thin‐walled structures [12], sandwich structures of hexagonal honeycomb thin‐walled tubes and micro‐perforated plates [13], micro‐perforated plate crossed‐partition thin‐walled tubes [14], chiral multi‐level nested thin‐walled tubes [15], among others. The weak coupling design approach has achieved multifunctional integration to some extent, but the independence of the functional units limits the further improvement of the overall structural performance. In contrast, the corresponding design method is strong coupling design, particularly designs based on lattice structures, which demonstrate greater potential. Lattice structures are composed of micro‐scale lattice unit cells arranged periodically at the macro scale according to certain rules. This multi‐scale structural characteristic provides outstanding design freedom, making them an ideal porous material for breaking through design freedom bottlenecks and improving equipment performance. The lightweight and porous nature of lattice structures is conducive to acoustic wave modulation, while efficient mechanical load‐bearing can be achieved through ingenious topology optimization design, providing natural advantages for multifunctional integrated design. Similar structural design forms include non‐integer‐dimensional architected metamaterial [16], interwoven dual‐phase lattice metamaterial [17, 18], bioinspired microlattice [19], and TPMS‐based composite microlattice [20]. Notwithstanding recent breakthroughs, contemporary architected materials predominantly relying on uniform strut, shell, or plate topologies often fail to incorporate the intricate micro‐features essential for significant viscous energy loss. Furthermore, these designs typically exhibit insufficient structural diversity to synergize broadband acoustic attenuation with concurrent mechanical robustness and thermal regulation.

Evolutionary adaptation has endowed turtles with an armor that functions as a highly efficient protective shield, optimized not only for mechanical load‐bearing but also for environmental wave modulation. Anatomically, the armor is characterized by a macroscopic curved skeletal geometry which enhances structural stiffness and deflects external impacts. Crucially, at the microscopic level, cross‐sectional analysis reveals that this curved shell encloses a sandwich configuration containing a core of distributed hollow cavities and trabecular networks [21, 22]. This hierarchical porosity plays a pivotal role in acoustic attenuation: as incident sound waves penetrate the shell, the interconnected cavities induce viscous friction and thermal loss within the pores, effectively dissipating acoustic energy. Concurrently, this porous architecture is instrumental in thermal regulation. The extensive surface area provided by the internal cavity network facilitates heat exchange, allowing for effective heat dissipation through convective transport mechanisms to maintain physiological homeostasis. Consequently, the curved turtle shell skeleton exemplifies a synergistic integration of impact protection, acoustic absorption, and thermal management, offering a blueprint for multifunctional lightweight design.

Addressing the inherent conflict between structural rigidity and acoustic porosity, we present a bioinspired multifunctional lattice metamaterial (BMLM) that emulates the arcuate cross‐section of turtle shells. This bioinspired topology, augmented by micro‐pores and strut‐diameter gradients successfully synergizes impact resistance with sound dissipation capabilities. Consequently, the BMLM exhibits a superior specific energy absorption of 55.7 kJ/kg, while simultaneously achieving an average sound absorption coefficient of 0.954, maintaining values above 0.9 across a continuous 2150–4250 Hz frequency band. Crucially, the architecture demonstrates exceptional acoustic robustness under compressive loading; even at 40% strain, the sound absorption coefficient remains exceeding 0.9 within a 2.1 kHz bandwidth. Beyond mechano‐acoustic duality, the interconnected open‐cell architecture ensures efficient fluid ventilation and thermal dissipation. This work establishes a unified framework for architected materials that demand high‐efficiency energy absorption, broadband noise mitigation and thermal management, offering a new paradigm for multifunctional engineering applications.

## 2 Turtle Shell‐Inspired Metamaterial Design

**FIGURE 1:**
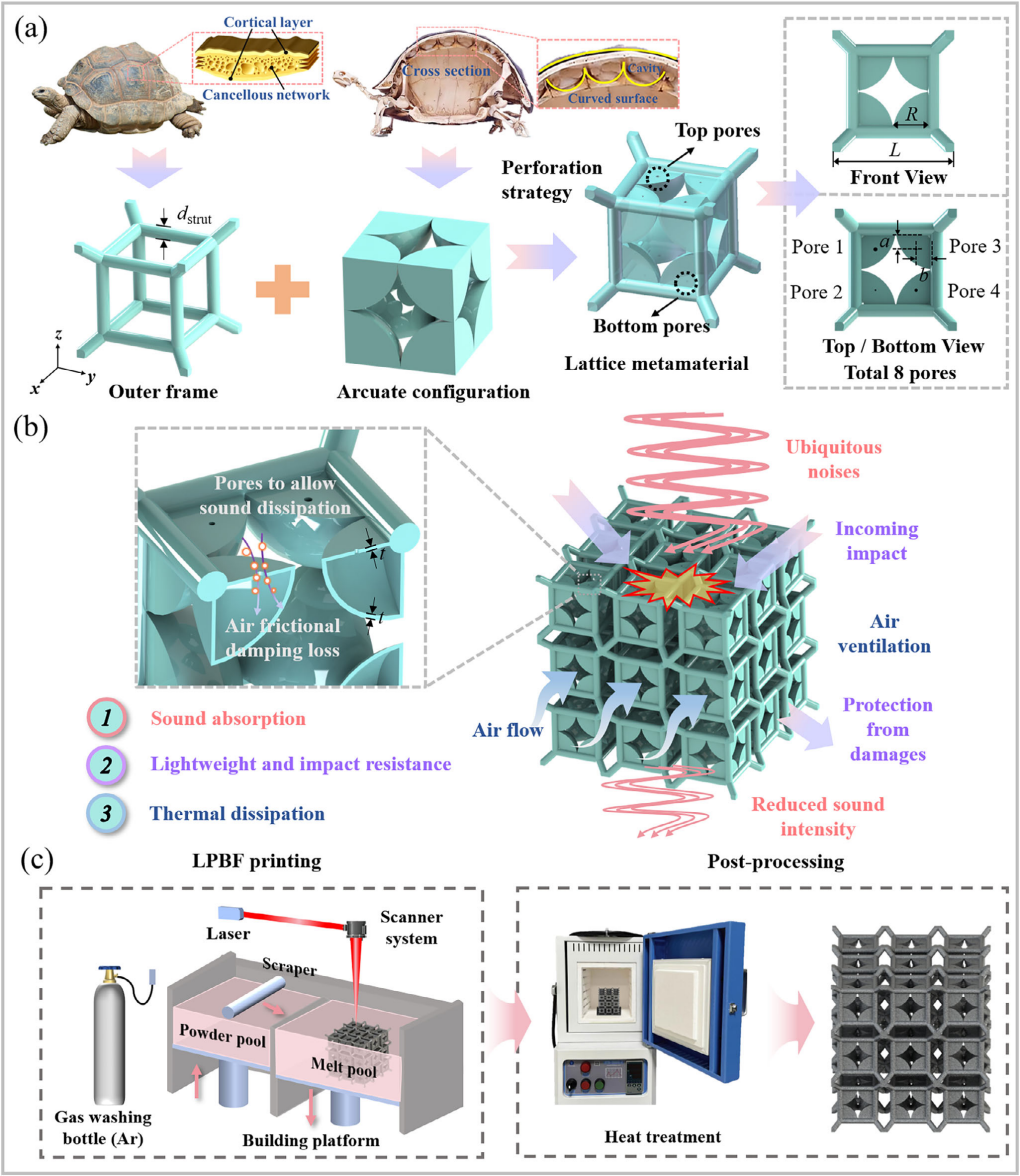
Overview of the lattice metamaterial design and fabrication. (a) Bioinspired design strategy for multifunctional lattice metamaterials. (b) Schematic illustration of noise and impact energy hazards, alongside a proposed multifunctional lattice architecture integrating impact resistance, noise mitigation, and ventilation with heat dissipation. (c) Fabrication of BMLM specimen via LPBF 3D printing.

Over millions of years of natural selection, turtles have evolved a highly sophisticated armor that strikes an optimal balance between lightweight construction and impact resistance under harsh environmental conditions. As illustrated in Figure 1a, the turtle shell exoskeleton features a hierarchical sandwich architecture composed of stacked curved layers. This curvature‐driven geometry plays a pivotal role in diffusing local stress concentrations, thereby endowing the structure with exceptional specific stiffness and energy dissipation capabilities. The resulting architecture is defined by both the curved surface geometry and the spatial arrangement and connectivity of the arcuate plate‐strut framework, interconnected pores, and internal void network. Here, this structural arrangement is referred to as the lattice topology, which governs the mechanical load‐transfer pathways, acoustic resonance/dissipation pathways, and fluid transport channels within the same architecture. The geometric parameters of a single curved surface are defined by the following function (in mm):

(1) $$x^{2}+y^{2}+z^{2}=R^{2}$$

Here *x*, *y*, and *z* represent the three directions of the coordinate axes as shown in Figures 1a and *R* denotes the radius of the sphere corresponding to the curved surface and *R* is taken as 10 mm in this work. The spherical surface generated by this function is then thickened to form a cell wall with a thickness *t* of 0.6 mm. Mechanically, this global curvature is introduced to exploit the arch effect, allowing localized bending loads to be redistributed as membrane‐dominated compressive stresses along the curved surface. Such stress redistribution mitigates local stress concentrations and provides the structural basis for the turtle shell‐inspired load‐bearing mechanism adopted in the proposed lattice. Additionally, each unit cell is formed by sealing the spherical shell with three orthogonal 0.6 mm‐thick flat plates, thereby creating a fully enclosed and isolated cavity. To ensure geometric stability of the lattice architecture, the outer frame of the lattice is supported by cylindrical elastic struts and eight inclined cylindrical elastic struts, whose inner extensions intersect at the geometric center of the lattice architecture. All cylindrical struts have a uniform diameter *d*_strut_ of 3.2 mm. The lattice unit cell occupies a cubic volume of *L* ^3^, where the side length *L* is 30 mm. The micro‐lattice structure defined in this manner is uniform and can be repeated periodically to form an array structure. The corresponding lattice architecture obtained from this microlattice array is illustrated in Figure 1b.

Notably, in addition to its excellent impact resistance, the interconnected void volume within the lattice architecture offers a versatile platform for tailoring acoustic functionalities. This design rationale is further rooted in the hierarchical sandwich‐like organization of the turtle shell, where a dense outer cortical layer encloses a porous internal cancellous network. Translating this biological layout into the metamaterial, the continuous outer frame of the unit cell serves as the stiff load‐bearing counterpart, while the engineered internal cavities and heterogeneous porous features mimic the cancellous network. This hierarchical mapping shifts the design from a homogeneous lattice to a functionally differentiated architecture, in which the outer framework preserves structural integrity and the internal void network enables acoustic attenuation. To embed an efficient acoustic damping mechanism into the architecture, pores are introduced on the outer surfaces of the quasi‐spherical cavities, thereby incorporating highly effective sound attenuation into the internal structure. In the top view, four micropores are distributed on the panel enclosing the upper cavities. The centers of these pores are positioned at distances *a* and *b* relative to the central axis of the strut, where *a* and *b* are both equal to *R*/2. Correspondingly, another set of four pores of the same size is arranged on the panels of the lower cavities. The quasi‐spherical domains inside the lattice act as compartmentalized acoustic resonators, and the pore locations are shown in Figure 1a. Based on the critical dissipation effect of subwavelength apertures and the coherent coupling theory of resonators, a highly efficient non‐uniform longitudinal arrangement is proposed in this work. In each transverse layer, the lattice structure is assigned identical pore diameters and layouts; along the longitudinal direction, however, layers with different geometric parameters are connected in series to form a hybrid resonant system that achieves broadband continuous sound absorption. In the mechanical characterization of this metamaterial, and without compromising its acoustic performance, the diameters of the micro‐lattice elastic struts between different layers are further optimized in a graded manner to enhance the overall structural mechanical properties. In this way, heterogeneity between the acoustic components and the mechanical components is introduced into the global configuration of the metamaterial. By maintaining a constant overall relative density and varying only the pore size and strut diameter of the microlattice unit cells, the layer‐dependent acoustic resonance behavior and the gradient design of energy absorption associated with the strut geometry are systematically investigated. Following the conceptual design of the BMLM, all specimens were 3D printed through the laser powder bed fusion (LPBF) technique, as schematically illustrated in Figure 1c.

## 3 Results and Discussion

### 3.1 Broadband Continuous Sound Absorption

**FIGURE 2:**
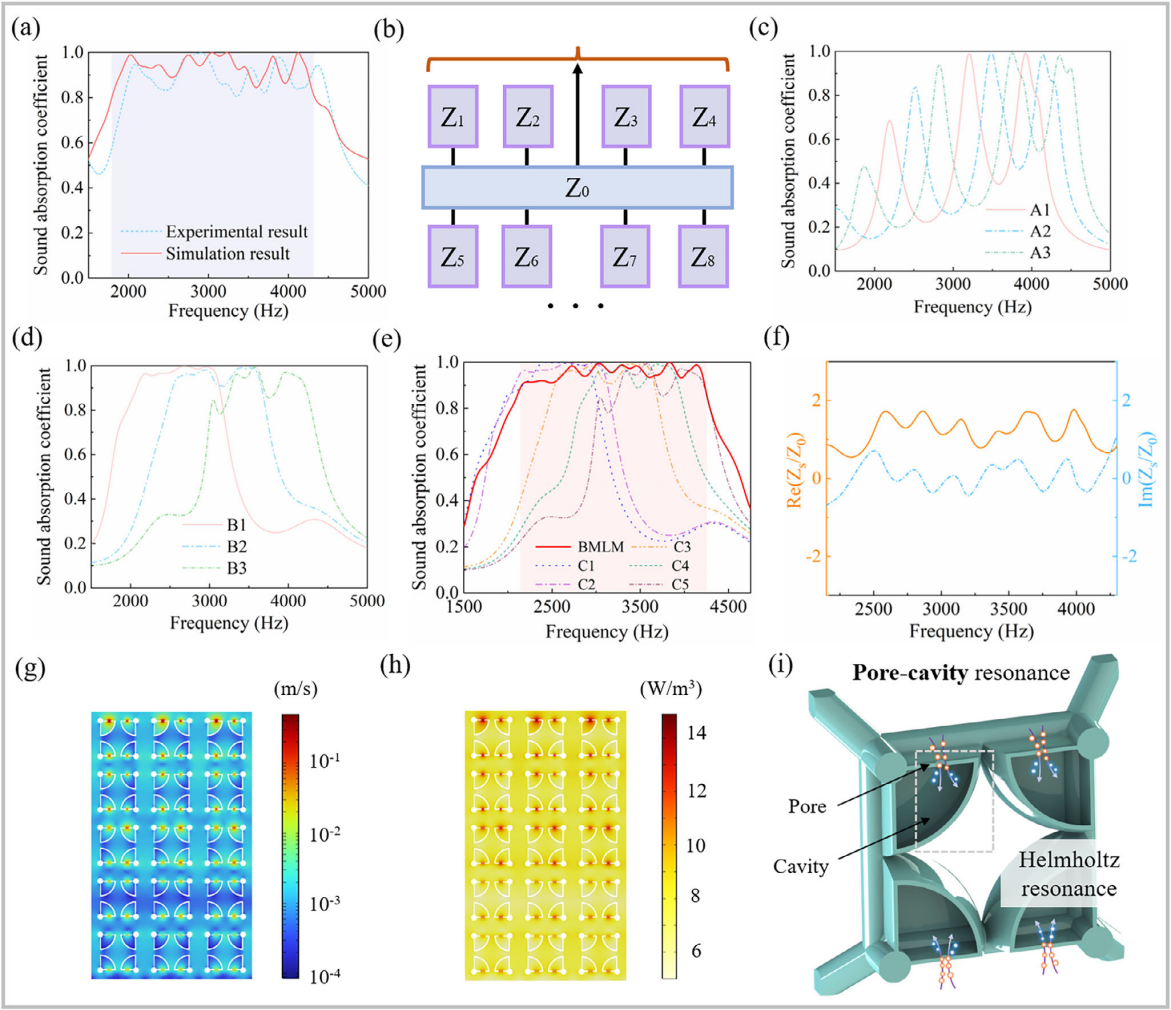
Overview of sound absorption performance. (a) Comparison between experimental and simulated sound absorption performances of the BMLM. (b) Acoustic impedance model based on the equivalent circuit method. Absorption coefficient curves of bio‐inspired lattice metamaterials with varying pore size distributions at an inter‐pore spacing of (c) 0.6 mm and (d) 0.2 mm. (e) Sound absorption coefficient curves and (f) corresponding acoustic impedance curves of the series‐assembled bio‐inspired lattice unit cells with varying pore size distributions. (g) Particle vibration velocity and (h) total viscous‐thermal energy density distribution of the BMLM at 3010 Hz. (i) Schematic illustrating the sound absorption mechanisms in the BMLM.

Sound absorption performance of our BMLM is investigated through both experiments, and finite element (FE) modelling through acoustical physics. For all sound absorption measurements, samples with five‐unit cells along sound incidence were utilized. As shown in Figure 2a, the representative experimentally measured sound absorption curve agrees well with the numerical result, verifying the accuracy of the FE model. Nevertheless, inherent discrepancies are observed due to surface roughness and geometric imperfections introduced by the 3D printing process. Within the broadband frequency range of 2150–4250 Hz, the average absorption coefficient reaches 0.954, and all measured data points exceed 0.9.

In the BMLM absorber, sound attenuation occurs primarily through viscous airflow passing through the subwavelength pores at resonance. The oscillatory motion of air within these apertures induces friction between the air and the pore walls, thereby converting the kinetic energy of the air molecules into heat and reducing the propagating acoustic energy. A small portion of the remaining energy is further dissipated at various geometric boundaries of the architecture. The broadband absorption mechanism arises from the serial arrangement of lattice layers over a wide frequency range, where each lattice unit incorporates a distinct combination of pores, collectively forming a hybrid resonant system. The good agreement between the simulation and experimental results for the BMLM structure demonstrates that the finite element approach can accurately predict the sound absorption performance of lattice metamaterials over different frequency bands.

#### 3.1.1 Effect of Pore Size Parameter

In designing this type of metamaterial, we first focus on sound absorption and analytically derive the acoustic impedance using the electro‐acoustic analogy as shown in Figure 2b. By evaluating each constituent component, the overall impedance of the metamaterial can be obtained. As noted from Figure 1, each unit cell consists of eight distinct resonators, in the form of an eighth of a spherical shell with perforations introduced. In the impedance calculation of the resonators, the effective acoustic impedance of an individual resonator *Z*_n_ (*n* = 0,1,2…,8) is composed of the perforation impedance *Z*_mn_ and the cavity impedance *Z*_cn_, specifically:

(2) $$Z_{n}=Z_{mn}+Z_{cn}$$

Under the condition that the acoustic wavelength is much larger than the cavity length, the sound pressure inside the entire cavity can be assumed to be spatially uniform. Accordingly, the cavity impedance of each resonator can be approximated as:

(3) $$Z_{cn}=-j\frac{S\rho_{0}c_{0}^{2}}{\omega V_{n}}$$

where *ρ*_0_ = 1.21 kg·m^−3^ and *c*_0_ = 343 m·s^−1^ are the air density and the speed of sound in air, respectively, *ω* = 2*πf* is the angular frequency with *f* being the excitation frequency, *S* = *L* ^2^ is the area of the incident sound field, and *V*_n_ denotes the cavity volume of the resonator.

The perforation impedance *Z*_mn_ can be calculated using the equations proposed by Maa [23]:

(4) $$Z_{mn}=\frac{j\omega \rho_{0}t}{\varphi }{\left[1-\frac{2J_{1}\left(\gamma \sqrt{-j}\right)}{\left(\gamma \sqrt{-j}\right)J_{0}\left(\gamma \sqrt{-j}\right)}\right]}^{-1}+j\frac{0.85\omega \rho_{0}d}{\varphi }+\frac{\sqrt{2}\eta_{0}\gamma }{\varphi d}$$

(5) $$\gamma =\frac{d}{2\sqrt{\frac{\eta_{0}}{\rho_{0}\omega }}}$$

where *φ* = *S*_an_/*S* is the perforation ratio (*S*_an_ is the cross‐sectional area of the pore). *γ* is the ratio of the pore radius to the viscous boundary layer thickness, and 𝜂_0_ is the dynamic viscosity of air. *J*_1_ represents the first kind Bessel function of order 1, while *J*_0_ corresponds to that of order 0. Here, *d* and *t* in Equations ((4), (5)) correspond directly to the sets of *d* and *t* shown in Figure 1a. *V*_n_ equals to the volume of a quadrant, given as $\frac{1}{6}\pi {\left(R-t\right)}^{3}$.

Based on the equivalent circuit approach, the overall acoustic impedance *Z_p_* of a single unit cell can be obtained and is given by:

(6) $$Z_{P}={\left(Z_{1}^{-1}+Z_{2}^{-1}+\cdots +Z_{8}^{-1}\right)}^{-1}$$

Subsequently, in series with the large resonant cavity, the total impedance *Z* of the BMLM is given by:

(7) $$Z=Z_{m0}+{\left({Z_{P}}^{-1}+{Z_{c0}}^{-1}\right)}^{-1}$$

Finally, the sound absorption coefficient, *α*, of the BMLM structure can be expressed as:

(8) $$\alpha =1-{\left|\frac{Z-\rho_{0}c_{0}}{Z+\rho_{0}c_{0}}\right|}^{2}$$

To investigate the effect of pore diameter parameters on the sound absorption performance of the BMLM unit cell, a series of analytical acoustic calculations were conducted. The pore diameter parameters of the unit cells are listed in Table S1. As shown in Figure 2c, as the pore diameter increases (at intervals of 0.6 mm), the absorption peak gradually shifts toward higher frequencies; however, the absorption peaks associated with each individual unit cell correspond only to discrete narrow frequency bands. Subsequently, to address this, the pore diameter parameters were adjusted to increase in increments of 0.2 mm as illustrated in Figure 2d. Due to resonance effects, the absorption curve demonstrates continuous broadband characteristics within the corresponding frequency range. In this band, the average sound absorption coefficient exceeds 0.9, and the coefficients across the specific frequency range are all above 0.9. Results show that as the pore diameter continues to increase, the absorption band shifts toward higher frequencies, while the overall absorption performance remains virtually unchanged.

In the BMLM unit cell, variations in pore diameter directly influence the propagation path of the sound waves and the associated energy dissipation, where the acoustic energy is converted into heat through friction, viscous effects, and thermal conduction, thereby affecting the sound absorption performance. By tuning the pore‐size parameters, the absorption behavior of each lattice layer can be enhanced within different frequency ranges. When these layers are then connected in a hierarchical series configuration, broadband sound absorption can be achieved.

#### 3.1.2 Effect of the Serial Coupling Effect

To investigate the effect of the series coupling effect of the lattice metamaterial on its sound absorption performance, five groups with continuously varying pore diameters and superior absorption performance were arranged in series. Each unit cell in the series was regarded as an independent absorber. The pore diameter parameters of the unit cells are listed in Table S1. As shown in Figure 2e, the sound absorption spectrum of the overall BMLM structure was obtained through simulation analysis. Results indicate that the whole BMLM exhibits an average absorption coefficient of 0.954, with values exceeding 0.9 across a continuous frequency range from 2150 Hz to 4250 Hz, validating the efficacy of the proposed design.

Furthermore, the physical mechanism of acoustic energy dissipation was elucidated through a coupled analysis of acoustic impedance matching and damping characteristics. As indicated by the sound absorption formula (Equation 7), the absorption coefficient exhibits characteristics typical of resonant absorbers, where each resonance peak corresponds to a specific set of impedance matching conditions. Maximum acoustic energy dissipation is achieved when the surface impedance of the BMLM structure nearly coincides with the characteristic impedance of air at the resonant frequency, i.e., Re(Z_s_/Z_0_) = 1 and Im(Z_s_/Z_0_) = 0. Here, the imaginary part characterizes the position of the resonant frequency, while the real part directly reflects the damping state. As shown in Figure 2f, perfect impedance matching is observed near the frequency *f*_1_ = 3840 Hz. Subsequently, the real part of the impedance rises continuously to a peak, then follows a downward trend, and finally fluctuates around 1.0 near the frequency *f*_2_ = 4090 Hz; this impedance mismatch explains the absorption valley observed in Figure 2e.

Moreover, to further investigate the sound absorption mechanism of the BMLM, Figure 2g, h depict the particle vibration velocity and viscous‐thermal dissipation distributions of the BMLM at 3010 Hz. Results indicate that peak particle velocity and viscous‐thermal dissipation are predominantly concentrated within the resonance pores. This phenomenon is attributed to the interplay between viscous friction and thermal dissipation mechanisms. Notably, viscous dissipation acts as the dominant energy dissipation pathway, exhibiting significantly higher efficiency compared to thermal dissipation (Figure 2i). In addition, the dissipation radiates downward in a diffusive manner, and different layers dominate the acoustic energy dissipation at different frequency bands. As the number of structural layers increases, the particle vibration velocity along the direction of sound propagation significantly decreases, particularly for particles exhibiting viscous behavior. This velocity attenuation induces a substantial gradient in air particle velocity, notably within the near‐field and far‐field regions of the resonance pores. Such discrepancies in particle velocity facilitate the formation of complex and dynamic flow fields within the confined regions of the resonance pores, thereby significantly influencing the aerodynamic characteristics of the system. Simultaneously, attributed to the siphon effect, air particles accumulate in the vicinity of the micro‐perforations [24]. Accompanied by intense molecular vibrations as they traverse the micropores, this process drives viscous‐thermal losses that convert acoustic waves into thermal energy. Consequently, the incident sound intensity is effectively attenuated.

Notably, the whole lattice architecture consists of five lattice layers with identical thickness but different porosities and acoustic impedances, each exhibiting distinct sound absorption characteristics. According to the propagation behavior of acoustic waves, the design of each layer can be tailored to induce different absorption and reflection responses in specific frequency bands. In addition, the multiple interfaces within the layer give rise to reflection of sound waves, thereby increasing the interaction between the waves and the architectures and enhancing the overall absorption efficiency. Moreover, the lattice structures at different layers may exhibit resonant behavior in particular frequency ranges. This cooperative behavior further strengthens the absorption of incident sound. During the parametric study of the sound absorption performance of the BMLM, numerical simulations combined with experimental validation clearly demonstrate the significant influence of these design parameters on the acoustic response, leading to an effectively hierarchical structure especially in terms of broadband near‐perfect absorption and mid‐high‐frequency noise control. Under multifunctional integration and dynamic deformation, the structure also exhibits a tunable response, which improves its feasibility for future practical applications.

### 3.2 Compression‐Resistant Deformation Behavior

#### 3.2.1 Deformation Mechanism

For the overall energy‐absorption performance of the BMLM, its crashworthiness is evaluated using the specific energy absorption (*SEA*) and energy absorption (*EA*) indices. Here, *EA* represents the total energy dissipated during the deformation of the structure, which can be obtained by integrating the load‐displacement response as follows:

(9) $$EA=\int_{0}^{d}F\left(x\right)\;\;dx$$

where *F(x)* is the instantaneous crushing force and *d* is the crushing distance. The *SEA* denotes the energy absorbed per unit mass of the structure and is defined as:

(10) $$SEA=\frac{EA}{m}=\frac{\int_{0}^{d}F\left(x\right)dx}{m}$$

where *m* is the total mass of the structure. A higher *SEA* value indicates a stronger energy absorption capability.

To further investigate the mechanical behavior, quasi‐static compression tests were conducted to evaluate the compressive characteristic of the BMLM. Firstly, a 2 × 2 × 2 BMLM, with a relative density of 24.5%, was adopted for the mechanical tests to reveal the structural deformation, while retaining the pore parameters corresponding to the acoustic components.

**FIGURE 3:**
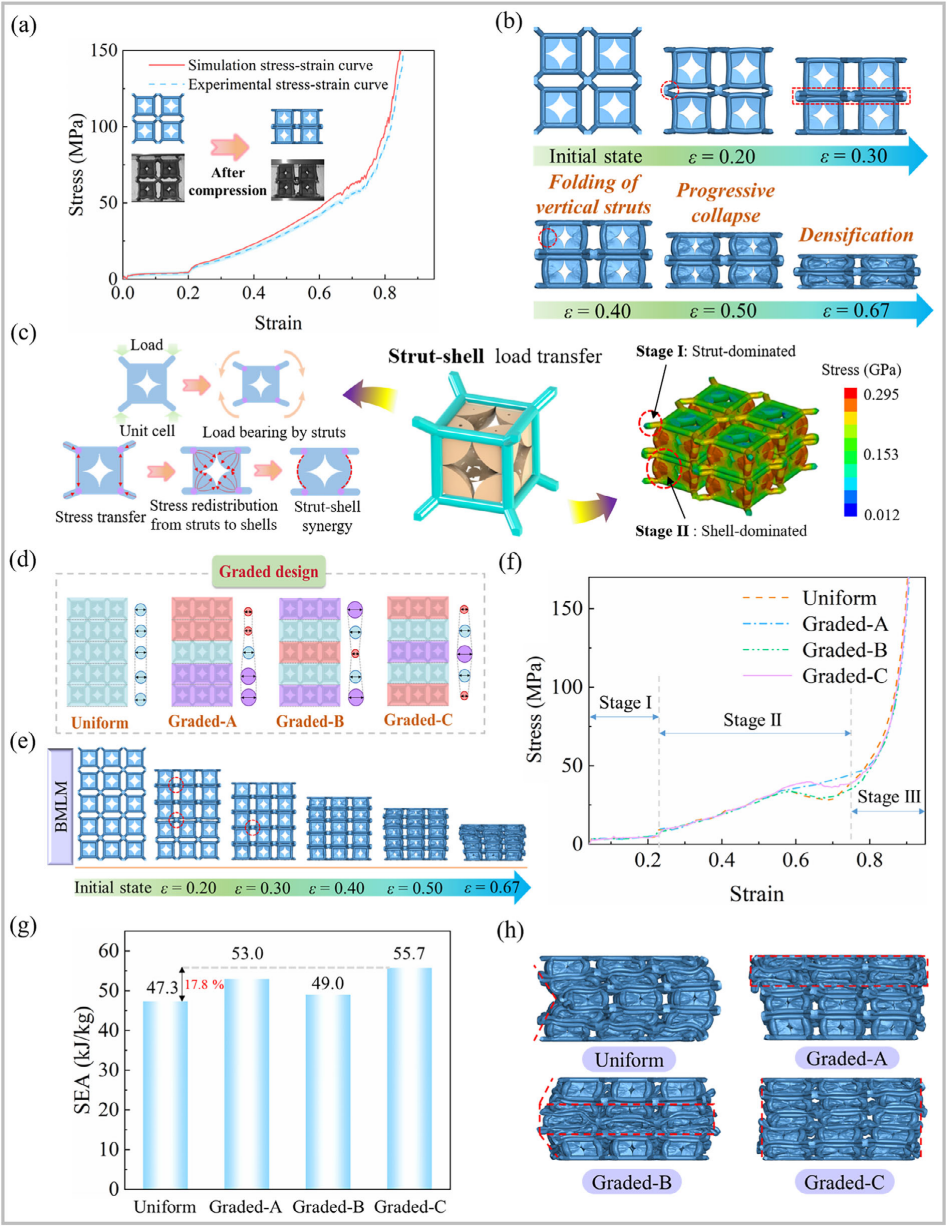
Mechanical performance and deformation analysis. (a) Comparison between experimental and simulation stress‐strain curves of the BMLM (2 × 2 × 2 array). (b) Deformation modes of the BMLM (2 × 2 × 2 array). (c) Schematic diagram of the strut‐shell load transfer path. (d) Gradient design strategy for the strut diameters of lattice metamaterials. (e) Deformation modes of the graded BMLM (3 × 3 × 5 array). (f) Comparison of compressive stress‐strain curves for gradient lattice metamaterials. (g) Comparison of SEA for the gradient lattice architectures. (h) Comparison of deformation modes in the latter part of Region II of the uniform and graded structures.

As shown in the comparison between the FE simulation and representative experimental stress‐strain curves (Figure 3a), the strong consistency between the simulation and experimental curves validates the fidelity of the FE model. Subsequently, as shown in Figure S8, a comparison between experimental and simulation curves for uniform and three kinds of graded structures provide further assessment of predictive accuracy of FE models (Section S5). Moreover, this favorable compression performance is reflected in its deformation behavior. Due to the presence of inclined supporting struts and arcuate acoustic cells, a distinct two‐stage plateau response is observed as shown in Figure 3b: during the initial deformation or under relatively mild impact, the BMLM can still maintain favorable acoustic performance, which is discussed in detail in Section 4.2. Once the supporting struts in the BMLM are fully crushed and failed, the arcuate shell‐based architecture takes over the second stage of impact protection and undergoes plastic deformation. At a compressive strain of 30%, the deformation mode reveals that outer inclined supporting struts resist deformation primarily through bending. As deformation progresses, the unique arcuate architecture, together with the connecting struts and orthogonal flat plates, provides smooth stress flows, effectively alleviates excessive stress concentrations, and enhances the overall structural strength. Overall, the arc‐shaped shell geometry plays a critical role in smoothing stress flows and mitigating stress concentrations typically found at nodal junctions. As shown in Figure 3c, the strut‐shell hybrid architecture creates a hierarchical load‐bearing network that guides cross‐scale stress redistribution. Localized stresses are effectively delocalized and redirected along the continuous strut‐shell pathways, facilitating an adaptive load transfer from local features to the global framework. Therefore, the progressive two stage deformation behaviors of the BMLMs clearly demonstrate the effectiveness of this bio‐inspired design, which endows the lattice metamaterial with outstanding sound absorption while simultaneously maintaining an efficient mechanical energy flow dissipation mechanism.

The proposed design combines an internal arcuate configuration with an outer frame. In order to further demonstrate the advantages of their integration, a comparative study was conducted using numerical simulations of outer frame structure, internal arcuate structure, and hybrid structure, respectively. The stress‐strain curves and deformation modes are shown in Figures S9 and S10. Results show that the mutual constraint between these two components triggers a synergistic enhancement effect, transitioning the failure mechanism into a stable and progressive collapse mode. In addition, the hybrid design achieves the most delayed densification among the three, with its SEA significantly increasing by 145.0% and 72.7% compared to the solely outer frame structure and internal arcuate structures, respectively.

#### 3.2.2 Strut‐Diameter Gradient Design

Following the investigation of the uniform lattice, we next explore the potential for enhancing energy absorption through functionally graded architectures (Figure 3d‐g). By maintaining a constant overall mass while simultaneously keeping the volume of the arc‐shaped cavities in each unit cell unchanged, three strut‐diameter gradient distribution modes for the lattice metamaterials were generated as shown in Figure 3d. Under each mode, the global deformation mode of the BMLM exhibits a distinct layer‐wise crushing sequence governed by the local strut diameter: layers with smaller strut diameters collapse first, followed by those with larger diameters, whereas the arcuate shell‐based architectures only undertake impact resistance after the supporting struts have exhausted their energy‐absorption capacity as shown in Figure 3e.

The stress‐strain curves and energy absorption data presented in Figure 3f,g demonstrate that introducing strut‐diameter gradation effectively enhances the energy‐absorption performance relative to the nongraded reference model. In particular, the best energy‐absorption behavior is obtained when the strut diameter gradually increases from the outer layers toward the inner layers, which also promotes a more stable deformation mode. In this optimal configuration, the SEA is improved by 17.8% compared with the nongraded design.

In this work, energy absorption efficiency *η* is used to determine the effective strain which presents the most optimal energy absorption capacity of structures [25], while the energy absorption efficiency *η* is defined as:

(11) $$W\left(\varepsilon \right)=\int_{0}^{\varepsilon }\sigma (\varepsilon )d\varepsilon$$

(12) $$\eta \left(\varepsilon \right)=\frac{W\left(\varepsilon \right)}{\sigma \left(\varepsilon \right)}$$

The densification strain *ε_D_*, which marks the onset of Stage III, is strictly defined as the strain corresponding to the absolute maximum value on the energy absorption efficiency‐strain curve. Based on the energy absorption efficiency‐strain curves of uniform and three kinds of graded structures shown in Figure S11, the onset of Stage III varies among the designs. The uniform structure reaches densification earliest at *ε_D_* = 0.69. In contrast, the graded structures effectively delay the densification process, with Graded‐A, Graded‐B, and Graded‐C initiating Stage III at *ε_D_* values of 0.79, 0.71, and 0.76, respectively. This demonstrates that the Graded‐A and Graded‐C design can postpone densification by approximately 14.5% and 10.1% compared to the uniform counterpart, thereby providing a substantially longer effective plateau stroke for energy absorption.

As illustrated in Figure 3g, the BMLM with different strut‐diameter gradients, but identical mass, exhibits markedly different energy‐absorption capabilities, confirming that diameter gradation is an effective strategy for enhancing the crashworthiness of lattice metamaterials when evaluated in terms of SEA. The improvement is especially pronounced with a contrast between the outer and inner layer strut diameters, where the inner layers adopt thicker struts, resulting in a substantial increase in SEA. As observed in the stress‐strain curves, the primary differences among the evaluated designs manifest prominently in the latter part of Region II (the plateau stage). To elucidate this behavior, a mechanistic analysis of their deformation modes is illustrated in Figure 3h. The structural stability during late‐stage compression strictly dictates the energy absorption capacity. For the uniform structure, the lack of a gradient design leads to unpredictable localized yielding, eventually culminating in a rightward tilting instability in the latter part of Region II. This buckling causes a drop in the latter part of Region II. Similarly, Graded‐B possesses the thinnest struts in its middle layer. This induces premature mid‐layer crushing followed by severe leftward tilting, which significantly compromises its stable deformation continuity. For Graded‐A (with increasing strut diameter top‐to‐bottom), while it initiates crushing at the top, the thicker bottom layers remain under‐deformed at equivalent macroscopic strains, indicating weaker material utilization. Conversely, Graded‐C demonstrates the most optimal deformation mechanism. The specific gradient design effectively guides a stable and progressive collapse. Throughout Region II, Graded‐C exhibits no lateral tilting or buckling. This exceptional structural stability ensures that the struts and plates fold and compact efficiently, allowing the structure to sustain a high and constant plateau stress, thereby maximizing the total energy absorbed.

### 3.3 Ventilation and Thermal Dissipation Characteristic

**FIGURE 4:**
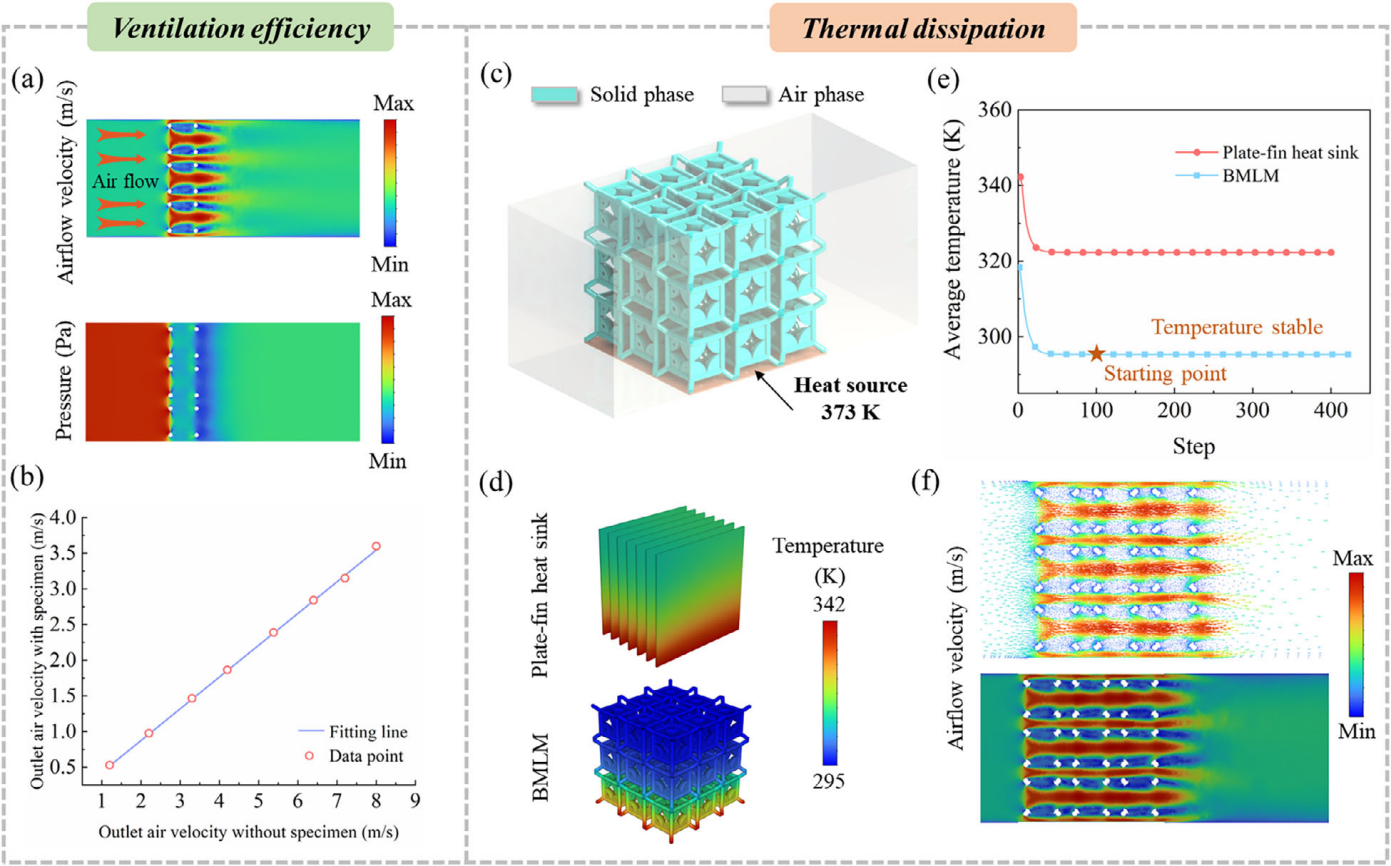
(a) Air flow field and pressure field distributions. (b) Ventilation effectiveness of the designed metamaterial. (c) Schematic of the BMLM with a fixed‐temperature heat source (373 K) applied to the bottom boundary of the air domain. (d) Temperature distribution contour plot of plate‐fin heat sink and BMLM. (e) Average temperature variation. (f) Airflow streamlines and velocity field distributions of the BMLM.

Ventilation and thermal dissipation efficiency were quantitatively evaluated via computational fluid dynamics (CFD) simulations. The internal flow field is represented by a vertical cross‐section in Figure 4a. Notably, a pronounced acceleration of airflow is observed within the lattice architecture. This phenomenon is governed by Bernoulli's principle, where the geometric constriction of the BMLM converts pressure potential into kinetic energy, resulting in a concomitant velocity surge and pressure drop. In addition, a comparative analysis of outlet velocities against varying inlet pressures were conducted for scenarios with and without the specimen (Figure 4b). Linear regression of the resultant dataset revealed a transmission coefficient of 0.45, confirming a ventilation efficiency of 45%.

Capitalizing on this high ventilation efficiency, the BMLMs exhibit exceptional thermal management capabilities, as elucidated by coupled fluid‐thermal multiphysics simulations. To assess convective heat transfer performance, a comparative analysis was conducted between BMLM and plate‐fin heat sink with the identical boundary conditions. With the base subjected to a constant heat source of 373 K (Figure 4c), a distinct vertical temperature gradient is established, driven by the interplay between solid‐phase conduction and ambient dissipation. Crucially, the introduction of a 5 m/s airflow acts as a catalyst for forced convection. The turbulent motion of the fluid medium within the complex interstitial spaces effectively intercepts the upward heat conduction along the lattice skeleton. As the fluid traverses the lattice, the large pressure drop across the constricted regions promotes local flow acceleration and increased instances of turbulence, thereby enhancing forced convective heat transfer and improving heat extraction from the structural features. The intensified convective transport steepens the axial temperature gradient, demonstrating the enhanced cooling capability of the BMLM (Figure 4d). As the surface temperature decreases, the thermal driving force (i.e., the temperature difference between solid and fluid) correspondingly diminishes, leading to a gradual reduction in heat transfer rate and an asymptotic approach toward thermal steady state.

The large specific surface area of the BMLMs inherently facilitates air permeation and circulation, thereby amplifying convective cooling efficiency. As illustrated in Figure 4e, which tracks the temperature evolution, the system achieves thermal equilibrium shortly after the introduction of steady airflow (approx. 100 simulation steps). Results show that the top surface temperature of the BMLM stabilizes at 295 K, whereas that of the plate‐fin heat sink stabilizes at 322 K. The internal velocity field, visualized in Figure 4f, offers further insight: the cross‐sectional view reveals a hybrid flow regime characterized by high‐speed streams within the central voids and complex shear flows around the strut‐plate junctions. This phenomenon effectively achieves thermal isolation between the heat source and the top surface, maintaining a top‐surface temperature significantly lower than that of traditional plate‐fin heat sink. Consequently, the BMLM integrates thermal management with protection by simultaneously dissipating heat from the source and shielding the top surface, making it an ideal candidate for applications requiring such dual‐functional thermal control.

## 4 Further Discussion

### 4.1 Discussion on Aperture Location

**FIGURE 5:**
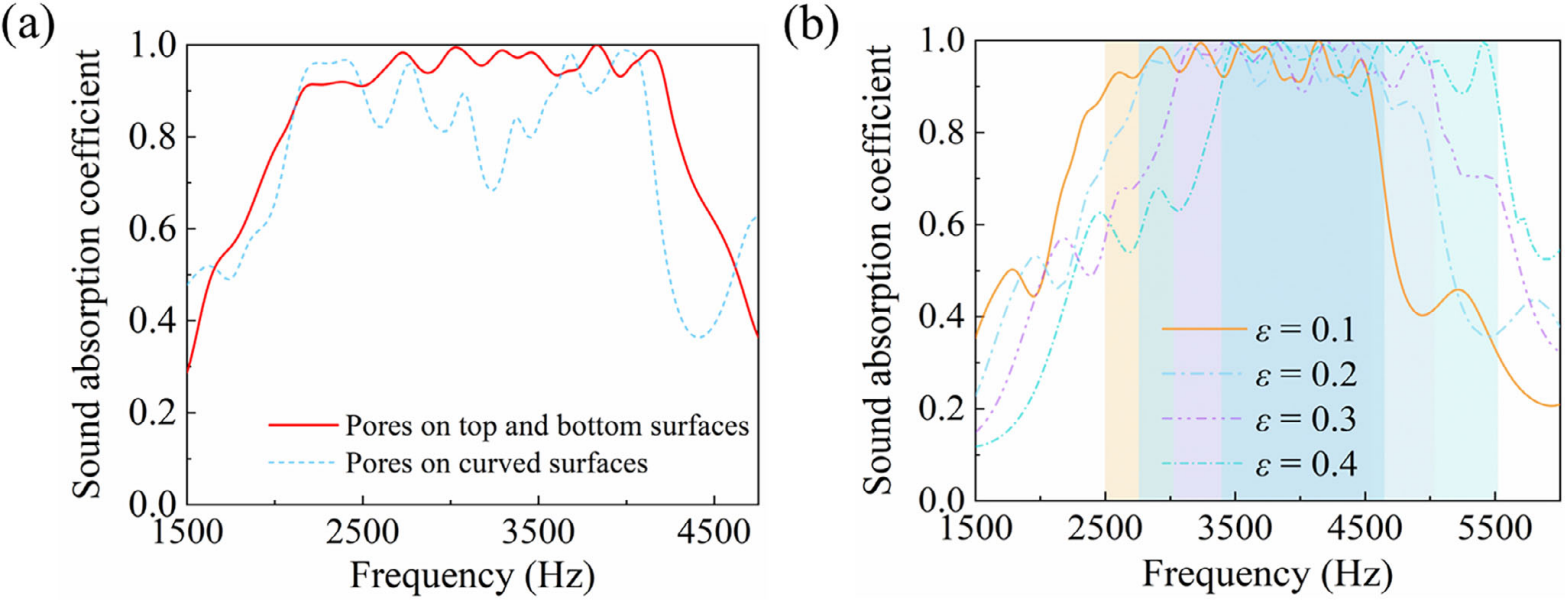
(a) Effect of pore position on the sound absorption performance of BMLMs. (b) Comparison of sound absorption performance under different compressive strains.

For most conventional sound‐absorbing devices, the pores are typically located along the line of incidence of the sound waves, which facilitates faster and more efficient energy dissipation through the apertures. Owing to its distinctive arcuate surface, the BMLM architecture allows the aperture positions to be reconfigured on the same air domain, which can effectively mitigate the adverse effects of water accumulation and dust deposition on the acoustic components in practical applications. To this end, while keeping all acoustic parameters unchanged, the pore locations of the BMLM are shifted from the top and bottom surfaces to the curved surface for a comparative study of sound absorption performance, as illustrated in Figure 5a. The center of each pore on the curved surface is located at the intersection point of the centerline of the support strut along the diagonal direction of the outer frame and the arcuate surface. The number of pores is eight in one unit cell. Due to the complete symmetry of the unit cell in the *z*‐direction, the two cavities in the vertical direction have the same pore diameter, resulting in four sets of different pore diameters in a single cell. The location and diameter of pores on the curved surface are shown in Figure S5 and Table S2. By adjusting the aperture position, the overall absorption band of the component can be maintained, although a reduction in acoustic performance is observed in certain frequency ranges, as summarized in Table S3.

Different pore locations modify the reflection and propagation patterns of sound waves inside the material and thus influence the absorption efficiency. Specifically, variations in aperture position affect the sound absorption through changes in the propagation path, multiple reflections, and impedance matching conditions. The altered incidence path leads to changes in the effective propagation distance and, consequently, in the absorption response. In addition, apertures placed at different locations can guide the waves into distinct reflection modes, thereby modifying the residence time of sound within the material. At the same time, shifting the pore positions can induce resonance enhancement in different frequency bands and thus alter the absorption capability. These results indicate that BMLM has the potential to maintain the sound absorption bandwidth while adjusting aperture positions, albeit at the cost of a partial reduction in acoustic performance metrics for specific application scenarios. Moreover, placing the apertures on the curved surface relaxes the constraint of a single preferred incidence direction, enabling further development of the omnidirectional sound‐absorbing capability of the BMLM and opening up new avenues for future inquiry.

### 4.2 Sound Absorption Performance After Compressive Deformation

From the perspective of the BMLM structural characteristics, its multifunctional design concept successfully integrates sound absorption, lightweight architecture, and impact resistance. In the present work, the BMLM exhibits favorable acoustic feedback under crushing‐type loading: even at different compressive ratios, it is able to maintain an extremely wide continuous absorption bandwidth. This behavior arises from a synergistic coupling effect induced by structural deformation, on the basis of which the sound absorption performance can be further enhanced.

When subjected to compressive deformation, the volume of the arcuate acoustic cells decreases, and this reduction in air‐domain volume occurs concurrently with the overall compression of the BMLM lattice architecture. This uniform deformation mode, inherent to lattice architectures, offers substantial potential for tuning the continuous absorption bandwidth: by controlling the global compression ratio of the lattice architecture, the high‐absorption frequency band can be shifted while maintaining a large sound absorption coefficient. In this work, as shown in Figure 5b and Table S4, the BMLM structure configured in a 3 × 3 × 5 array maintains a high continuous absorption bandwidth, a high average absorption coefficient, and nearly perfect absorption (all measured points exceeding 0.9) over a range of compression ratios.

With increasing compressive strain, the mechanical response of the lattice metamaterial evolves. During the first stage of compression (crushing of the supporting struts), the porosity of the BMLM does not decrease, and the overall geometry of the acoustic architecture essentially preserves its original form. At this stage, the spacing between different layers is reduced, but before the compression strain reaches 20%, the structure still maintains broadband continuous near‐perfect absorption, with the corresponding absorption band shifting toward higher frequencies. When the compression strain reaches 40%, the continuous absorption band shifts more markedly to higher frequencies, while the average sound absorption coefficient remains at a high level. This is because moderate compression leads to a more compact structure, increasing the interaction path length between sound waves and the architecture, which enhances energy dissipation, thereby improving the absorption of high‐frequency sound. These findings indicate that proposed BMLM can enhance the sound absorption performance of lattice metamaterials after compressive deformation, particularly in applications involving broadband mid‐high‐frequency continuous noise control.

## 5 Benchmarking and Future Research

**FIGURE 6:**
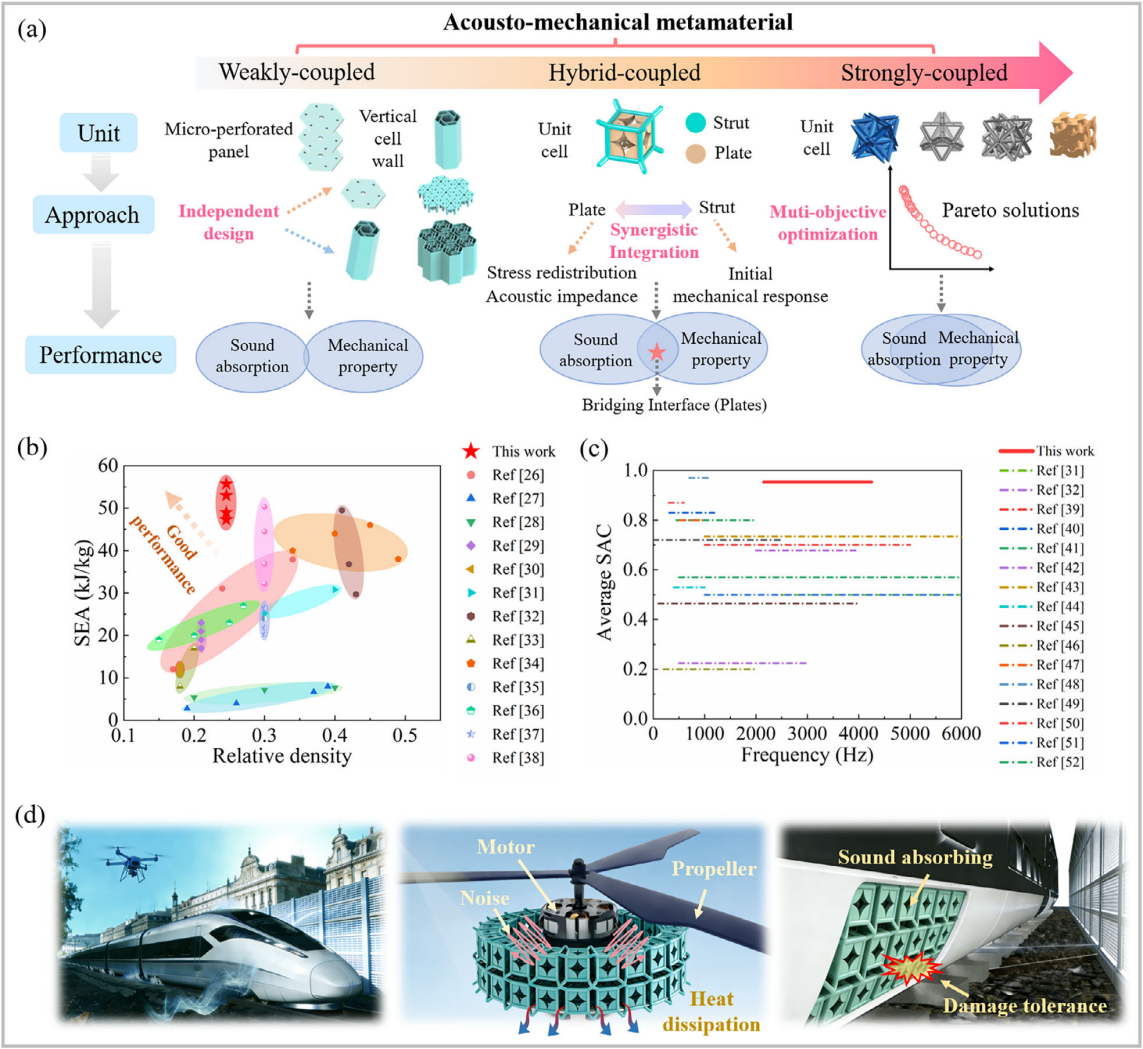
Research framework of acousto‐mechanical metamaterials. (a) Summary of the weakly‐coupled, hybrid‐coupled, and strongly‐coupled mechanism design approaches for manipulating sound‐absorbing and mechanical properties, revealing their distinctions in terms of unit design, research approach, and final performance outcomes. Comparison of the (b) specific energy absorption and (c) sound absorption coefficient of different lattice metamaterials. (d) Potential applications of the multifunctional BMLM, exemplified using transport system.

Despite the surging interest in multifunctional metamaterials that integrate sound‐absorbing and mechanical capabilities, a unified design philosophy remains largely underdeveloped. Here, we propose a framework based on the degree of functional coupling, positioning metamaterials along a continuum rather than as strictly discrete categories (Figure 6a). At one end are weakly‐coupled systems, where acoustic and mechanical roles are largely segregated, for instance, resonant acoustic elements integrated within a load‐bearing skeleton. In such architectures, geometric parameters governing acoustic impedance can be tuned with minimal influence on mechanical stiffness, enabling near independent optimization. At the opposite end are strongly‐coupled systems, in which a single topological network simultaneously governs both stress transmission and acoustic wave propagation. In these architectures, geometric parameters of the unit cell concurrently modulate mechanical deformation and acoustic impedance, rendering independent tuning fundamentally impossible. A multi‐objective optimization workflow is required to resolve the inherent conflicts in strongly‐coupled designs, identifying the Pareto frontier for optimal performance. These include most of the basic lattice metamaterials, including trusses and triply periodic minimal surface lattices.

In this research our BMLM exemplifies a hybrid case: strut‐dominated regions primarily dictate the initial mechanical response, while the plates act as bridging interfaces that simultaneously influence stress redistribution and acoustic impedance. These plate elements therefore serve as pivotal coupling nodes between the two physical domains, giving rise to controlled but not fully inseparable functional interaction. Implementing this strategy, the BMLMs demonstrate that structural synergy can be engineered to satisfy specific demands, such as merging wide‐frequency absorption with robust mechanical plateaus.

The uniqueness of this design is reflected in its performance advantages compared to existing literatures [26, 27, 28, 29, 30, 31, 32, 33, 34, 35, 36, 37, 38, 39, 40, 41, 42, 43, 44, 45, 46, 47, 48, 49, 50, 51, 52]. To highlight its innovativeness, the comparison of similar structures is shown in Ashby charts. Two key indicators were selected including SEA and average sound absorption coefficient to plot Ashby charts. As shown in (Figure 6b), the BMLM exhibits exceptional energy absorption characteristics compared with other metal lattice structures, foams, and cellular composites. Under the same relative density conditions, the BMLM exhibits the highest SEA. Similarly, when SEA remains the same, the BMLM achieves a lighter weight with lower relative density. In addition, the BMLM exhibits superior average sound absorption coefficient compared with other lattice configurations (Figure 6c). Within the same frequency band of 2150 to 4250 Hz, the structure exhibits the highest average sound absorption coefficient, reaching 0.954. With a relatively wide bandwidth of 2100 Hz, it demonstrates significant sound absorption advantages.

To demonstrate the practical engineering versatility of BMLM, we propose targeted integration strategies for noise‐sensitive environments as visualized in (Figure 6d). In the realm of unmanned aerial vehicles, the intense acoustic signature stemming from high‐speed propellers and compact motors critically compromises both urban quietness and military stealth capabilities. To mitigate this, BMLM architectures can be conformally applied to encapsulate motor housings, serving a dual function: attenuating broadband noise while enabling efficient thermal management via their permeable porous topology. Parallelly, for high‐speed rail systems where aerodynamic turbulence and wheel‐rail contact induce problematic low‐to‐mid frequency vibrations BMLM modules can be strategically embedded within underbody fairings, interior linings, or highway noise barriers. This deployment effectively dissipates acoustic energy, thereby significantly elevating passenger acoustic comfort and curbing environmental noise pollution. Beyond their acoustic capabilities, these modules possess the structural robustness to withstand high‐velocity foreign object impacts. This dual‐function deployment effectively dissipates acoustic energy while ensuring mechanical durability, thereby significantly elevating passenger acoustic comfort and safety.

## 6 Conclusion

In this work, a turtle shell‐inspired lattice metamaterial was designed and fabricated to achieve simultaneous mechanical, acoustic, and thermal functionalities within a unified architected framework. By integrating arcuate plate‐strut geometries with hierarchical microporosity, the proposed architecture enables topology‐driven coupling of energy dissipation, sound attenuation, and convective heat transfer. Experimental results demonstrate that the BMLM achieves outstanding multifunctional performance, combining a high specific compressive energy absorption of 55.7 kJ/kg with excellent broadband acoustic behavior. The structure exhibits an average sound absorption coefficient of 0.954 and maintains near‐unity absorption (>0.9) across a continuous frequency band from 2150 to 4250 Hz. Complementing acoustic and mechanical properties, the BMLM offers effective thermal management. The open‐cell topology facilitates efficient fluid flow, resulting in a marked cooling effect that underscores the superior heat dissipation capability of the BMLM. Second, the BMLM exhibits remarkable acoustic robustness under complex service conditions. Strategic placement of apertures on the arcuate surfaces mitigates performance degradation caused by blockage or dust accumulation. Notably, the sound absorption coefficient remains above 0.9 even at compressive strain of 40%, highlighting the structural stability in harsh mechanical environments. The BMLM fills a critical gap in current lattice metamaterials by offering a synergistic combination of broadband noise reduction, high impact resistance, and thermal regulation. These attributes provide a valuable framework for the design of next‐generation multifunctional metamaterials for engineering applications.

## 7 Experimental Section

### 7.1 Fabrication

All BMLM specimens were fabricated through LPBF 3D printing using an EOS M290 system equipped with a Yb fiber laser, with AlSi10Mg employed as the feedstock material. The laser operated at a power of 325 W, with a layer thickness of 30 µm and a scanning speed of 1400 mm/s. Prior to the LPBF process, the build chamber was purged with high‐purity argon gas to create an inert atmosphere. The argon purging process was maintained until the residual oxygen concentration inside the chamber dropped below 0.1%. This inert environment was kept throughout the manufacturing process to prevent the oxidation of the metal powder and the melt pool. After fabrication, the specimens were wire‐cut from the build plate, and any residual powder was carefully removed. The heat treatment regime consisted of solution treatment at 525°C for 2 h followed by water quenching, and subsequent artificial aging at 175°C for 12 h followed by water cooling. The fabrication process is schematically shown in Figure 1c.

### 7.2 Sound Absorption Testing

The acoustic performance of the BMLM specimens was characterized using a two‐microphone impedance tube setup in accordance with ISO 10534‐2 (Figure S1). Each specimen was rigidly mounted in a sample holder with an inner diameter of 29 mm. The measurement system provides a valid frequency range from 0.8 kHz to 6.3 kHz. The BMLM specimens were enclosed by a cylindrical adapter ring to ensure a tight fit within the tube; this outer ring served solely as a mechanical support and geometric adapter, and its influence on the acoustic measurements was considered negligible. All reported sound absorption coefficients were obtained by averaging three independent datasets. Detailed procedures were described in Section S2.

### 7.3 Mechanical Property Measurement

The mechanical response of the BMLM specimens was evaluated by quasi‐static compression tests using a Shimadzu universal testing machine (Figure S1). The samples were subjected to uniaxial quasi‐static compressive loading with the loading surfaces aligned parallel to the LPBF build direction. The velocity of the upper platen was set to 0.9 mm/min, which corresponds to a strain rate of 0.0005 s^−1^. Each BMLM specimen was placed at the center of the loading platen and compressed by the displacement of a rigid upper platen until densification was achieved. During testing, the deformation evolution of the structure was recorded using a digital image correlation (DIC) system (VIC‐2D). To ensure data reliability, all quasi‐static compression results were obtained by averaging three repeated tests.

### 7.4 Numerical Simulation

For the acoustic characterization, a finite element (FE) model of the metamaterial was constructed in COMSOL Multiphysics and discretized using a structured mesh. A perfectly matched layer (PML) was added on the incident side to absorb sound waves propagating along the negative *z*‐direction of the background pressure field (Figure S2). The pressure acoustics module was employed, with the interior region specified as an air domain subjected to a background pressure field. A thermos‐viscous boundary impedance condition was applied to accurately capture thermal‐viscous losses at the solid‐fluid interfaces. To ensure consistency with the experiments, the physical properties of the air in the model were set identical to the theoretical values used in the analytical calculations. Considering a maximum frequency of 5000 Hz, the mesh size in the acoustic domain was constrained to be smaller than one‐sixth of the corresponding wavelength, and further refined in the thermos‐viscous regions to be smaller than the thickness of the thermos‐viscous boundary layer. For the energy‐absorption analysis, quasi‐static compression simulations were carried out in LS‐DYNA (via ANSYS). The metallic lattice structure was modeled using an elastic‐plastic material model (MAT_024), and the stress‐strain response of AlSi10Mg was defined according to the curve shown in Figure S4. Detailed modeling procedures are described in Section S3.

## Author Contributions

X.W. and X.L. conceptualized the study and conducted the formal analysis. X.W., S.W., B.C., and X.L. developed the methodology. X.W. and S.W. were responsible for software development and validation. X.W., B.C., and X.L. conducted the investigation. X.W. wrote the original draft. B.C. and X.L. reviewed and edited the manuscript and supervised the project.

## Funding

This work was supported by National Natural Science Foundation of China under Grant No. 12502137, Natural Science Foundation of Liaoning Province under Grant No. 2025‐BS‐0445, and Science and Technology Talent Innovation Policy Plan of Dalian under Grant No. 2024RQ006.

## Conflicts of Interest

The authors declare no conflict of interest.

## Supporting information




**Supporting File**: adma74125‐sup‐0001‐SuppMat.docx.

## Data Availability

The data that support the findings of this study are available from the corresponding author upon reasonable request.

## References

[1] S. Yu , W. Guo , Z. Zhou , Y. Li , and J. Qiu , “Rough‐Endoplasmic‐Reticulum‐like Hierarchical Composite Structures for Efficient Mechanical‐Electromagnetic Wave‐Energy Attenuation,” Advanced Functional Materials 34, no. 14 (2024): 2312835, 10.1002/adfm.202312835.

[2] H. Pei , H. Yang , N. Zhang , et al., “Moth‐Wing‐Inspired Multifunctional Metamaterials,” Advanced Materials 38, no. 9 (2025): 15350, 10.1002/adma.202515350. PMC12902595 41399959

[3] C. Liu , L. Jiang , H. Liu , et al., “Bio‐inspired Composite Design with Variable‐stiffness Curvilinear Helicoidal Layup for Enhanced Impact Resistance,” Composites Part B: Engineering 309 (2026): 113078, 10.1016/j.compositesb.2025.113078.

[4] X. Li and Z. Li , “The Cuttlebone Blueprint for Multifunctional Metamaterials: Design Taxonomy, Functional Decoupling, and Future Horizons,” Advanced Functional Materials (2026): 30551, 10.1002/adfm.202530551.

[5] X. He , G. Li , L. Zhang , et al., “Bio‐inspired Material‐structure‐function Integrated Additive Manufacturing of Al‐based Metamaterials with Surpassing Energy Absorption,” Science Advances 11, no. 46 (2025): aea0430, 10.1126/sciadv.aea0430. PMC12617464 41237224

[6] Y. Xu , Y. Huang , H. Yan , et al., “Sunflower‐pith‐inspired Anisotropic Auxetic Mechanics from Dual‐gradient Cellular Structures,” Matter 6, no. 5 (2023): 1569–1584.

[7] H. Zhang , Y. Li , Z. Lin , Z. Zhang , D. Hu , and Z. Yang , “Excellent Mechanical Properties of a Novel Double‐diagonal Reinforced Mechanical Metamaterial with Tunable Poisson's Ratios Inspired by Deep‐sea Glass Sponges,” Materials & Design 250 (2025): 113628, 10.1016/j.matdes.2025.113628.

[8] J. W. Chua , T. Li , B. W. Chua , X. Yu , and W. Zhai , “Customisable Sound Absorption Properties of Functionally Graded Metallic Foams,” Journal of Materials Science & Technology 108 (2022): 196–207, 10.1016/j.jmst.2021.07.056.

[9] S. Sakamoto , Y. Maruyama , K. Yamaguchi , and K. Ii , “Experiment and Estimation of the Sound Absorption Coefficient for Clearance of Corrugated Honeycomb,” The Journal of the Acoustical Society of America 145, no. 2 (2019): 724–733, 10.1121/1.5089427. 30823784

[10] S. Xie , S. Yang , C. Yang , and D. Wang , “Sound Absorption Performance of a Filled Honeycomb Composite Structure,” Applied Acoustics 162 (2020): 107202, 10.1016/j.apacoust.2019.107202.

[11] K. Shi , D. Hu , D. Li , and G. Jin , “Sound Absorption Behaviors of Composite Functionally Graded Acoustic Structure under Hydrostatic Pressure,” Applied Acoustics 211 (2023): 109474, 10.1016/j.apacoust.2023.109474.

[12] Z. Li , X. Wang , K. Zeng , et al., “Unprecedented Mechanical Wave Energy Absorption Observed in Multifunctional Bioinspired Architected Metamaterials,” NPG Asia Materials 16 (2024): 45, 10.1038/s41427-024-00565-5.

[13] Z. Ren , Y. Chen , M. Chen , X. Yuan , and D. Fang , “A Compact Multifunctional Metastructure for Low‐frequency Broadband Sound Absorption and Crash Energy Dissipation,” Materials & Design 215 (2022): 110462, 10.1016/j.matdes.2022.110462.

[14] J. Fan , B. Song , L. Zhang , et al., “Structural Design and Additive Manufacturing of Multifunctional Metamaterials with Low‐frequency Sound Absorption and Load‐bearing Performances,” International Journal of Mechanical Sciences 238 (2023): 107848, 10.1016/j.ijmecsci.2022.107848.

[15] X. Wang , R. Qin , J. Lu , M. Huang , X. Zhang , and B. Chen , “Laser Additive Manufacturing of Hierarchical Multifunctional Chiral Metamaterial with Distinguished Damage‐resistance and Low‐frequency Broadband Sound‐absorption Capabilities,” Materials & Design 238 (2024): 112659, 10.1016/j.matdes.2024.112659.

[16] Z. Guo , Z. Lei , K. Zeng , et al., “Non‐integer‐dimensional Architected Materials Enabling Synergistic Acoustic, Mechanical, and Fluid Coupling,” Materials Horizons 13, no. 2 (2025): 748–762, 10.1039/D5MH01768H. 41183361

[17] Z. Li , K. Zeng , Z. Guo , et al., “All‐in‐One: an Interwoven Dual‐Phase Strategy for Acousto‐Mechanical Multifunctionality in Microlattice Metamaterials,” Advanced Functional Materials 35, no. 20 (2025): 2420207, 10.1002/adfm.202420207.

[18] Z. Li , X. Wang , Z. Wang , et al., “Emerging Acousto‐mechanical Metamaterials: from Physics‐guided Design to Coupling‐driven Performance,” Materials Today 89 (2025): 151–171, 10.1016/j.mattod.2025.06.029.

[19] Z. Li , X. Wang , X. Li , Z. Wang , and W. Zhai , “New Class of Multifunctional Bioinspired Microlattice with Excellent Sound Absorption, Damage Tolerance, and High Specific Strength,” ACS Applied Materials & Interfaces 15, no. 7 (2023): 9940–9952, 10.1021/acsami.2c19456. 36655583

[20] J. W. Chua , X. Li , and W. Zhai , “Design, Multiscale Modelling, and Experimental Characterisation of TPMS‐based Composite Lattices with Enhanced Sound Absorption,” Composite Structures 370 (2025): 119437, 10.1016/j.compstruct.2025.119437.

[21] B. Achrai and H. D. Wagner , “Micro‐structure and Mechanical Properties of the Turtle Carapace as a Biological Composite Shield,” Acta Biomaterialia 9, no. 4 (2013): 5890–5902, 10.1016/j.actbio.2012.12.023. 23271040

[22] H. Rhee , M. F. Horstemeyer , Y. Hwang , H. Lim , H. El Kadiri , and W. Trim , “A Study on the Structure and Mechanical Behavior of the Terrapene Carolina Carapace: A Pathway to Design Bio‐inspired Synthetic Composites,” Materials Science and Engineering: C 29, no. 8 (2009): 2333–2339, 10.1016/j.msec.2009.06.002.

[23] D.‐Y. Maa , “Potential of Microperforated Panel Absorber,” The Journal of the Acoustical Society of America 104 (1998): 2861–2866, 10.1121/1.423870.

[24] C. Liu , J. Wu , K. Lu , Z. Zhao , and Z. Huang , “Acoustical Siphon Effect for Reducing the Thickness in Membrane‐type Metamaterials with Low‐frequency Broadband Absorption,” Applied Acoustics 148 (2019): 1–8, 10.1016/j.apacoust.2018.12.008.

[25] Y. Xiang , T. Yu , and L. Yang , “Comparative Analysis of Energy Absorption Capacity of Polygonal Tubes, Multi‐cell Tubes and Honeycombs by Utilizing Key Performance Indicators,” Materials & Design 89 (2016): 689–696, 10.1016/j.matdes.2015.10.004.

[26] X. Zhang , S. Yan , X. Xie , Y. Li , C. Wang , and P. Wen , “Multi‐dimensional Hybridized TPMS with High Energy Absorption Capacity,” International Journal of Mechanical Sciences 273 (2024): 109244, 10.1016/j.ijmecsci.2024.109244.

[27] H. Zhang , D. Hu , H. Peng , W. Yuan , and Z. Yang , “In‐plane Crushing Behavior and Energy Absorption of Sponge‐inspired Lattice Structures,” International Journal of Mechanical Sciences 274 (2024): 109328, 10.1016/j.ijmecsci.2024.109328.

[28] W. Xu , C. Zhou , H. Zhang , Z. Liu , and P. Zhu , “A Flexible Design Framework for Lattice‐based Chiral Mechanical Metamaterials Considering Dynamic Energy Absorption,” Thin‐Walled Structures 203 (2024): 112108, 10.1016/j.tws.2024.112108.

[29] X. Ma , C. Gu , Y. Wang , J. Shen , and X. Wang , “Design, Fabrication and Mechanical Properties of Multi‐dimensional Graded‐amplitude Gyroid Structures,” Thin‐Walled Structures 210 (2025): 113002, 10.1016/j.tws.2025.113002.

[30] Y. Tian , H. Wang , Z. Chen , Q. Wu , and S. Yin , “Design Principles for Dual‐phase Lattice Cylindrical Tubes with Excellent Energy Absorption Capability,” Composite Structures 359 (2025): 119015, 10.1016/j.compstruct.2025.119015.

[31] Z. Yu , L. Chen , C. Zhang , et al., “A Multifunctional Heterogeneous Structure Inspired by Shell Brick‐mud Structure: Balancing Noise Reduction and Mechanical Performance,” Composites Science and Technology 256 (2024): 110765, 10.1016/j.compscitech.2024.110765.

[32] H. Wang , K. Wang , P. Ma , and X. Fan , “The Energy Absorption Characteristics and Sound Absorption Behavior of in Situ Integrated Aluminum Lattice Structure Filled Tubes,” Advanced Engineering Materials 26 (2024): 2401686, 10.1002/adem.202401686.

[33] X. Wang , X. Li , Z. Li , Z. Wang , and W. Zhai , “A Ribbed Strategy Disrupts Conventional Metamaterial Deformation Mechanisms for Superior Energy Absorption,” Virtual and Physical Prototyping 19 (2024): 2337310, 10.1080/17452759.2024.2337310.

[34] X. Wang , Z. Li , J. Deng , et al., “Unprecedented Strength Enhancement Observed in Interpenetrating Phase Composites of Aperiodic Lattice Metamaterials,” Advanced Functional Materials 35 (2025): 2406890, 10.1002/adfm.202406890.

[35] C. Bonatti and D. Mohr , “Smooth‐shell Metamaterials of Cubic Symmetry: Anisotropic Elasticity, Yield Strength and Specific Energy Absorption,” Acta Materialia 164 (2019): 301–321, 10.1016/j.actamat.2018.10.034.

[36] Z. Li , W. Zhai , X. Li , X. Yu , Z. Guo , and Z. Wang , “Additively Manufactured Dual‐functional Metamaterials with Customisable Mechanical and Sound‐absorbing Properties,” Virtual and Physical Prototyping 17 (2022): 864–880, 10.1080/17452759.2022.2085119.

[37] M. Zhao , Z. Li , J. W. Chua , C. H. Lim , and X. Li , “Enhanced Energy‐absorbing and Sound‐absorbing Capability of Functionally Graded and Helicoidal Lattice Structures with Triply Periodic Minimal Surfaces,” International Journal of Minerals, Metallurgy and Materials 30 (2023): 1973–1985, 10.1007/s12613-023-2684-8.

[38] X. Li , X. Yu , J. W. Chua , H. P. Lee , J. Ding , and W. Zhai , “Microlattice Metamaterials with Simultaneous Superior Acoustic and Mechanical Energy Absorption,” Small 17 (2021): 2100336, 10.1002/smll.202100336. 33984173

[39] J. Feng , J. Qiao , Q. Xu , G. Zhang , and L. Li , “Accelerated Design of Acoustic‐mechanical Multifunctional Metamaterials via Neural Network,” International Journal of Mechanical Sciences 287 (2025): 109920, 10.1016/j.ijmecsci.2025.109920.

[40] X. Liu , J. Wu , J. Niu , W. Li , and C. Liu , “Vibro‐acoustic Helmholtz Absorber with Soft Wall for Broadband Sound Absorption,” International Journal of Mechanical Sciences 289 (2025): 110083, 10.1016/j.ijmecsci.2025.110083.

[41] J. W. Chua , W. Zhai , and X. Li , “Elucidating Structure‐property Relationships for Optimization of Plate Lattice Sound Absorbers,” Materials & Design 253 (2025): 113801, 10.1016/j.matdes.2025.113801.

[42] L. Zhao , C. Dong , S. Li , et al., “Novel Design of Skin Construction on the Sound Absorption of Honeycomb Sandwich Panels,” Composite Structures 356 (2025): 118870.

[43] X. Li , X. Yu , M. Zhao , Z. Li , Z. Wang , and W. Zhai , “Multi‐Level Bioinspired Microlattice with Broadband Sound‐Absorption Capabilities and Deformation‐Tolerant Compressive Response,” Advanced Functional Materials 33 (2023): 2210160, 10.1002/adfm.202210160.

[44] H.‐Z. Li , J.‐S. Yang , Q. Liu , et al., “A Novel Sandwich Structure for Integrated Sound Insulation and Absorption,” International Journal of Mechanical Sciences 279 (2024): 109526, 10.1016/j.ijmecsci.2024.109526.

[45] C. Patil , R. Ghorpade , and R. Askhedkar , “Investigation into the Sound Absorptivity of Perforated Panels with Tapered Hole Geometries Coupled with Polyurethane Foam,” International Journal on Interactive Design and Manufacturing (IJIDeM) 19 (2025): 1849–1868, 10.1007/s12008-024-01864-5.

[46] C. Zhang , W. Tong , H. Qin , et al., “Multi‐modal Resonance of Topological Hybrid Graphene Foam for Enhanced Acoustic Absorption,” Chemical Engineering Journal 503 (2025): 158560, 10.1016/j.cej.2024.158560.

[47] M. Liang , H. Wu , S. Chen , Z. Fu , R. Zhang , and Z. Xing , “Simulated and Experimental Research on Highly Efficient Sound Absorption of Low‐Frequency Broadband Acoustic Metamaterial Based on Coiled‐up Space,” Advanced Engineering Materials 25 (2023): 2301221, 10.1002/adem.202301221.

[48] R. Qu , J. Guo , Y. Fang , S. Zhong , and X. Zhang , “Broadband Quasi‐perfect Sound Absorption by a Metasurface with Coupled Resonators at both Low‐ and High‐amplitude Excitations,” Mechanical Systems and Signal Processing 204 (2023): 110782, 10.1016/j.ymssp.2023.110782.

[49] L. Li , F. Yang , Y. Jin , et al., “Multifunctional Hybrid Plate Lattice Structure with High Energy Absorption and Excellent Sound Absorption,” Materials & Design 241 (2024): 112946, 10.1016/j.matdes.2024.112946.

[50] X. Li , S. Ding , X. Wang , S. L. A. Tan , and W. Zhai , “Recipe for Simultaneously Achieving Customizable Sound Absorption and Mechanical Properties in Lattice Structures,” Advanced Materials Technologies 10 (2024): 2400517, 10.1002/admt.202400517.

[51] Z. Hu , J. Ding , S. Ding , et al., “Machine Learning—Enabled Inverse Design of Shell‐based Lattice Metamaterials with Optimal Sound and Energy Absorption,” Virtual and Physical Prototyping 19 (2024): 2412198, 10.1080/17452759.2024.2412198.

[52] L. Chen , C. Wang , H. Ji , and J. Qiu , “Design and Analysis of a Microlattice Structure for Enhanced Broadband Sound Absorption,” Applied Acoustics 235 (2025): 110681, 10.1016/j.apacoust.2025.110681.

