# Supplementary material for: Topology‐Orchestrated Multi‐physical Energy Control in Turtle Shell‐Inspired Metamaterials
Source: Adv Mater. 2026 Jul 16;38(47):e74125. doi: 10.1002/adma.74125 (PMC13496050; doi:10.1002/adma.74125)
Supplement: Supplementary file 1 [file ADMA-38-e74125-s001.docx]

Supporting Information

Topology-Orchestrated Multiphysical Energy Control in Turtle Shell-Inspired Metamaterials

Xi Wang, Shiyi Wang, Bingzhi Chen*, and Xinwei Li*

X. Wang, S. Wang, B. Chen

Key Laboratory of Railway Industry on Safety Service Key Technologies for High-speed Train

Zhan Tianyou Honors College

Dalian Jiaotong University

Dalian 116028, China
E-mail: chenbingzhi06@hotmail.com

X. Li.

Newcastle University in Singapore

Faculty of Science, Agriculture, and Engineering

Newcastle University

Newcastle upon Tyne NE1 7RU, UK

E-mail: [xinwei.li@newcastle.ac.uk](mailto:xinwei.li@newcastle.ac.uk)

**Section S1. Fabrication of specimens**

The specimens were fabricated using an EOS M290 LPBF system (EOS GmbH, Germany), equipped with a 400 W fiber laser. The machine features a build volume of 250 mm×250 mm×325 mm. The printing process was conducted in an inert argon atmosphere. AlSi10Mg alloy powder was selected as the feedstock material for the bio-inspired multifunctional lattice metamaterial (BMLM) due to its favorable combination of low density, excellent castability, high thermal conductivity, and superior corrosion resistance. Regarding the processing parameters, the laser power *P* was set to 325 W, the scanning speed *v* to 1400 mm/s, the laser exposure time to 200 μs, the layer thickness to 30 μm, and the hatch spacing to 140 μm. Subsequently, a post-process heat treatment was performed under an argon atmosphere to relieve residual stresses and optimize the material performance. The regimen consisted of solution treatment at 525°C for 2 hours followed by water quenching, and subsequent artificial aging at 175°C for 12 hours followed by water cooling.

**Section S2. Experimental testing**

The establishment of acoustic impedance tube testing and compression test are illustrated in Figure S1. It is worth noting that while the mechanical compression tests were conducted using aluminum alloy samples to evaluate their energy absorption capacities, the acoustic testing samples for the impedance tube were fabricated using photosensitive resin via SLA 3D printing. According to the classical rigid-frame porous material theory, the sound absorption mechanism in such metamaterials is governed by the visco-thermal dissipation of air within the interconnected cavities. Given that the acoustic impedance of both resin and aluminum is orders of magnitude higher than that of air, both materials act as virtually rigid boundaries for incident sound waves. Therefore, the acoustic performance is strictly dictated by the topological geometry of the internal fluid channels rather than the intrinsic modulus of the solid skeleton. Utilizing SLA printed resin samples ensures higher surface finish and dimensional precision (exactly 29 mm in diameter), strictly avoiding boundary air leakage in the impedance tube, thereby yielding highly accurate acoustic properties. The CAD models representing the four strain conditions alongside photographs of the actual 3D-printed samples used for the acoustic tests are shown in Figure S2. The experimental and simulation SAC curves of acoustic cylindrical specimens with a diameter of 29 mm under different strains are shown in Figure S6 and Figure S7. Results show that the experimental SAC curves under different strains agree well with the numerical ones, confirming the accuracy of the established acoustic FE models.

**Figure S1.** Establishment of the (a) acoustic testing setup, (b) mechanical testing setup.

**Figure S2.** CAD models representing the four strain conditions alongside photographs of the actual 3D-printed samples used for the acoustic tests.

**Section S3. Finite element modeling**

As depicted in Figure S3, the experimental setup was emulated by positioning the model between two rigid plates. The bottom plate was secured to restrict all degrees of freedom, while the upper plate was programmed to move downward at a uniform velocity of 1 m/s in the *z*-direction, with constraints applied to movements in other directions. In the simulation, the contact between BMLM and the rigid walls was simulated using *CONTACT_AUTOMATIC_SURFACE_TO_SURFACE, and self-contact within BMLM was simulated using *CONTACT_AUTOMATIC_SINGLE_SURFACE, with a friction coefficient of 0.15. In addition, an elastic-plastic material model was employed within LS-DYNA for AlSi10Mg material, parameterized by the experimental stress-strain curves illustrated in Figure S4. Both rigid plates were defined as rigid bodies with an element size of 1 mm, and the lattice model was discretized through Tetra solid elements with element size of 0.2 mm, as recommended by the mesh convergence analysis. Although a higher speed of 1 m/s was used in the numerical analysis in comparison with to the experimental setup to enhance computational efficiency, the simulation is regarded as quasi-static due to the kinetic energy remaining consistently less than 5% of the internal energy throughout the compressive process.

**Figure S3.** Establishment of finite element models: (a) compression simulation model, and (b) sound absorption simulation model.

**Figure S4.** Engineering and true stress-strain curves of the AlSi10Mg alloy.

**Section S4. Sound absorption characteristic**

**Table S1** Pore size distributions of BMLMs with various configurations.

| Structural  configuration | *d*_hole1_ (mm) | *d*_hole2_ (mm) | *d*_hole3_ (mm) | *d*_hole4_ (mm) |
| --- | --- | --- | --- | --- |
| A1 | 0.4 | 1.0 | 1.6 | 2.2 |
| A2 | 0.6 | 1.2 | 1.8 | 2.4 |
| A3 | 0.8 | 1.4 | 2.0 | 2.6 |
| B1 | 0.8 | 1.0 | 1.2 | 1.4 |
| B2 | 1.2 | 1.4 | 1.6 | 1.8 |
| B3 | 1.6 | 1.8 | 2.0 | 2.2 |
| C1 | 0.6 | 0.8 | 1.0 | 1.2 |
| C2 | 0.8 | 1.0 | 1.2 | 1.4 |
| C3 | 1.2 | 1.4 | 1.6 | 1.8 |
| C4 | 1.4 | 1.6 | 1.8 | 2.0 |
| C5 | 1.8 | 2.0 | 2.2 | 2.4 |

**Table S2** Pore size distributions located on the curved surfaces with various configurations.

| Structural  configuration | *d*_pore1_ (mm) | *d*_pore2_ (mm) | *d*_pore3_ (mm) | *d*_pore4_ (mm) |
| --- | --- | --- | --- | --- |
| Cell 1 | 0.6 | 0.8 | 1.0 | 1.2 |
| Cell 2 | 0.8 | 1.0 | 1.2 | 1.4 |
| Cell 3 | 1.2 | 1.4 | 1.6 | 1.8 |
| Cell 4 | 1.4 | 1.6 | 1.8 | 2.0 |
| Cell 5 | 1.8 | 2.0 | 2.2 | 2.4 |

**Table S3** Effect of pore opening position on the sound absorption performance of BMLM structures.

| Pore opening  position | Continuous absorption bandwidth (Hz) | Average absorption coefficient | Absorption performance metrics |
| --- | --- | --- | --- |
| Top and bottom surfaces | 2150-4250 | 0.954 | >0.9 (100%) |
| Arcuate surfaces | 2150-4250 | 0.871 | >0.8 (72.8%) |

**Table S4** Sound absorption characteristics of BMLM at different compressive strains.

| Strain *ε* | Continuous absorption bandwidth (Hz) | Average absorption coefficient | Sound absorption coefficient（*α*>0.9） |
| --- | --- | --- | --- |
| *ε*= 0.1 | 2500-4550 | 0.961 | 100% |
| *ε*= 0.2 | 2750-4650 | 0.956 | 100% |
| *ε*= 0.3 | 3030-5030 | 0.959 | 97.5% |
| *ε*= 0.4 | 3390-5510 | 0.958 | 94.4% |

**Figure S5.** Schematic diagram of pore distribution on the curved surface of unit cell.

**Figure S6.** SAC curves of acoustic cylindrical specimens with a diameter of 29 mm under different strains.

**Figure S7.** Comparison of experimental and simulation sound absorption coefficient curves of acoustic cylindrical specimens with a diameter of 29 mm under different strains.

**Section S5. Mechanical characteristic**

The densification strain *ε_D_*, which marks the onset of Stage III, is strictly defined as the strain corresponding to the absolute maximum value on the energy absorption efficiency-strain curve. After calculation, the four kinds of structures do not share the same initial position at Stage III. Among them, the strain of the uniform structure is 0.69, the strain of the Graded-A structure is 0.79, the strain of the Graded-B structure is 0.71, and the strain of the Graded-C structure is 0.76. In fact, this variation is a crucial advantage of gradient design. Compared to the strain of the uniform structure (0.69), Graded-A and Graded-C significantly delays the densification process up to a strain of 0.79 and 0.76. This is because the progressive collapse of the graded layers extends the plateau region, providing a much longer effective stroke for energy absorption. Three repeated compression experiments were conducted for each structural configuration to verify the reproducibility. The stress-strain curves exhibit excellent consistency.

**Figure S8.** Comparison of experimental and simulation stress-strain curves of uniform and three kinds of graded structures.

A comparative study was conducted using numerical simulations of outer frame structure, internal arcuate structure, and hybrid structure, respectively. The stress-strain curves and deformation modes are shown in Figure S9 and Figure S10. From the perspective of deformation modes, the solely outer frame structure is prone to global buckling, whereas the internal arcuate structure is highly susceptible to structural instability due to lacking the necessary support from vertical struts, leading to the earliest onset of densification. Upon integration, the hybrid structure exhibits a distinct two-stage deformation sequence, characterized by the successive response of the struts and the arcuate plates. The mutual constraint between these two components triggers a synergistic enhancement effect, transitioning the failure mechanism into a stable and progressive collapse mode. Consequently, the hybrid design achieves the most delayed densification among the three, with its SEA significantly increasing by 145.0% and 72.7% compared to the solely outer frame structure and internal arcuate structures, respectively. Additionally, since the outer frame is a strut-based structure, its standalone sound absorption and thermal resistance are negligible due to the lack of sufficient visco-thermal surface area. Therefore, the internal arcuate configuration is identified as the primary functional carrier for sound absorption and heat regulation. The role of the outer frame in this hybrid integration is to provide the necessary acoustic boundaries and mechanical performance to maintain the internal functional architecture under service loads.

**Figure S9.** Stress-strain curves and SEA values of outer frame structure, internal arcuate structure, and hybrid structure, respectively.

**Figure S10.** Deformation modes of outer frame structure, internal arcuate structure, and hybrid structure, respectively.

**Figure S11.** Comparison of energy absorption efficiency-strain curves of uniform and three kinds of graded structures.
